# In Support of Simian Polyomavirus 40 VP4 as a Later Expressed Viroporin

**DOI:** 10.1128/mSphere.00187-20

**Published:** 2020-03-18

**Authors:** Robert Daniels, Daniel N. Hebert

**Affiliations:** a Division of Viral Products, Center for Biologics Evaluation and Research, Food and Drug Administration, Silver Spring, Maryland, USA; b Department of Biochemistry and Molecular Biology and Program in Molecular and Cellular Biology, University of Massachusetts, Amherst, Amherst, Massachusetts, USA

**Keywords:** nonenveloped virus, simian virus 40, viral release, viroporin

## Abstract

Simian virus 40 VP4 was discovered in 2007 as a later expressed viral protein initiated from a downstream Met on the VP2/VP3 transcript. VP4’s role as a viroporin involved in viral release was supported in a series of additional articles that characterized the ability of VP4 to associate with and permeabilize biological membranes. This commentary is our response to the perspective from Henriksen and Rinaldo (mSphere 5:e00019-20, 2020, https://doi.org/10.1128/mSphere.00019-20) that challenges the existence of SV40 VP4.

## COMMENTARY

Progress in science benefits from the critical evaluation of data, as independent assessments can often help to shape the subsequent interpretations, hypotheses, or models. In this issue, Henriksen and Rinaldo (1) have presented a perspective that questions our previous work showing that simian polyomavirus 40 (SV40) expresses a later protein termed VP4, which contributes to SV40 lytic release by likely acting as a viroporin (2). Our responses to the concerns voiced in this perspective are detailed below.

To start, it is appropriate to provide a brief summary of the conception and the rationale of the study. At the time, one research line in the lab focused on investigating why the minor SV40 structural proteins, VP2 and VP3, are both essential since these are encoded by overlapping, in-frame, coding regions (3, 4). We were particularly intrigued by the observation that multiple hydropathy prediction algorithms assigned hydrophobic stretches to VP2 and VP3 that are generally associated with membrane proteins because it was becoming clear that SV40 penetration likely occurs at the endoplasmic reticulum (ER) membrane (3, 5). Based on these potential links, we designed a strategy to assess the ability of *in vitro*-translated [^35^S]Cys/Met-labeled VP2 and VP3 to integrate into ER-derived membranes using ultracentrifugation and autoradiography (3). Upon analyzing the total translation products on a Tris-Tricine gel, we unexpectedly observed a smaller, ∼14-kDa product. This led us to scan the VP2 coding region for a reading frame that corresponded in size with this product, and one was identified with a potential ATG initiation codon that was in-frame with VP2 and VP3. We then mutated this potential Met initiation codon to Ile (M228I in VP2), which was chosen because of the side chain similarity to Met, and the band was no longer observed, confirming that the ∼14-kDa *in vitro*-synthesized product initiated from this ATG. These *in vitro* translation-based results were not interpreted as a line of biological proof. Instead, they were included in the study to provide the context for the discovery process. The NetStart1.0 prediction scores were also included because the values suggested that this Met could act as an initiation site, and like the hydropathy analysis tools, these are only predicted values, not proof, and require experimental validation.

In our previous work showing that VP2 and VP3 were important for viral entry (3, 4), one of the key approaches was to examine BS-C-1 cells transfected with mutant SV40 genomes, as this allowed us to differentiate the entry and release processes and to demonstrate that VP2- and VP3-deficient genomes can rescue one another in *trans*. Therefore, we applied this same methodology to determine if the SV40 genome expressed the 14-kDa protein that was observed using *in vitro* translations. The initial experimental design involved the analysis of mutant SV40 genomes where the initiation codons were mutated for both VP2 and VP3 (ΔVP2/3), for all three proteins (ΔVP2/3/4), or for just the downstream in-frame ATG (ΔVP4). Based on the resulting immunoblot assays using a rabbit polyclonal antiserum that was raised against full-length VP3 provided by Ariella Oppenheim’s lab, a distinct band at the appropriate molecular weight was observed only when the VP4 initiation codon was present, leading us to conclude that the SV40 genome can express VP4. With the genome that does not express VP4 (ΔVP4), faint protein bands were observed in the low-molecular-weight region of the gel, indicating these could represent VP2 and VP3 proteolytic products. However, when the ΔVP2/3 genome was used, a clear VP4 band was observed together with similar faint bands challenging the conclusion that all products in this region arise from VP2 and/or VP3 proteolysis.

During the immunoblotting experiments, we observed that the VP4 band was not readily apparent using the following: (i) postnuclear supernatants obtained from cells lysed with 1% of the detergent NP-40, (ii) lysates from cells that had been harvested by trypsinization, (iii) SDS-PAGE gels that had been transferred to a polyvinylidene difluoride (PVDF) membrane for long periods of time, or (iv) Tris-glycine SDS-PAGE gels (2–4). However, VP4 was visualized by immunoblotting at late infection times (∼3 days) when the cells were harvested by scraping, the insoluble nuclear fraction (obtained from treating cells with 1% NP-40) was further solubilized using 2% SDS, and the sample buffer containing samples was sonicated on ice and then incubated at 25°C for 30 min. These observations served as indicators that VP4 localized to the nucleus and likely remained in the cell following lysis. Thus, it was not surprising that VP4 was observed in the nuclear membrane by confocal immunofluorescence microscopy using the ΔVP2/3 mutant genome (2), a profile also observed for enhanced green fluorescent protein (eGFP)-VP4 or VP4-myc constructs (6, 7).

The potential role for VP4 in cell lysis is supported by several lines of evidence. From our work, we were never able to observe significant levels of untagged VP2, VP3, or VP4 in mammalian cells when they were expressed using standard mammalian expression plasmids, indicating that these proteins could be toxic or have some viral requirement for expression (R. Daniels and D. N. Hebert, unpublished results). As negative results are not a helpful argument, we turned to Escherichia coli in an effort to produce recombinant VP2/3/4 proteins for biochemical studies. To avoid steric problems introduced by large tags such as glutathione *S*-transferase (GST), which is commonly used to express VP2 and VP3, we used a pET vector with a small C-terminal 6×His tag, and to our surprise, almost no protein was expressed in the soluble or insoluble fraction of E. coli Rosetta 2(DE3)pLysS cells. In fact, the E. coli culture expressing VP3-His lysed shortly after the addition of the induction agent (2, 4). We then determined that VP3 without the VP4 initiation codon did not lyse E. coli and that VP4 alone was not enough for bacterial lysis (2). Hygromycin accessibility experiments confirmed that in addition to lysis, the E. coli cells were also permeabilized by VP3 when the initiation codon for VP4 was present, suggesting the two viral proteins may work together. In support of this hypothesis, VP2/3/4 were shown to form oligomers with recombinant GST-VP3. Finally, compared to wild type (WT), we saw a delay in viral lysis when the WT and ΔVP4 infections were initiated by transfection, indicating that the mutant has a problem with release as the viral genome transfection approach bypasses the entry viral stages of entry and penetration.

Reinforcing the potential viroporin role of VP4 in cell lysis, several follow-up studies demonstrated that recombinant VP4 was also capable of permeabilizing membranes from a variety of sources, including red blood cells, Cos-7 cells, and liposomes (7–10). Furthermore, the release of encapsulated fluorescent markers of defined sizes from liposomes demonstrated that VP4 created pores with an inner diameter of 1 to 5 nm (10). The membrane disruption properties of VP4 combined with its late expression time that coincides with virus-induced cell lysis support the model that was initially proposed where VP4 acts as a viroporin to induce cell lysis and the release of the nonenveloped virus SV40. While it is interesting to speculate about BK polyomavirus (BKPyV), which also possesses a downstream Met, we did not examine a potential VP4 in this particular virus. However, the Atwood and DiMaio labs showed data for a putative VP4 BKPyV that also are in line with what we observed for SV40 and supportive for a role in viral release (11).

A main concern of Henriksen and Rinaldo was that they were unable to visualize VP4 after viral infection, an issue also raised by Nakanishi and colleagues (12). There are a number of properties of VP4 that can complicate its visualization and identification, including that (i) it is a small protein that runs off commonly used Tris-glycine gel systems and requires shorter transfer times for immunoblotting; (ii) it is not soluble in Triton-based detergents, which include NP-40, resulting in it frequently being discarded with nuclear membrane fractions; (iii) it is expressed at a later time after transfection (3 days) with the viral genome; (iv) it is hydrophobic and like other membrane proteins is prone to aggregate upon heating in sample buffer; (v) epitope exposure is expected to be minimal due to its hydrophobic property, which can hinder isolation by immunoprecipitation; (vi) cells must be isolated by mechanical means for analysis as it is trypsin sensitive in lysed cells; and (vii) not all antisera are equally reactive against an antigen, especially one with few epitopes. This last problem is important to mention since there was a period after this publication when we were unable to visualize VP4. When we inquired with the antibody source at this later time, we were notified that the antigen had been changed to a region that largely corresponded to the portion of VP3 that did not contain VP4. After our query a number of years ago, the company eventually corrected the description for this antibody. The variability of antibodies makes it essential to show that a particular antigen is capable of recognizing a target using the specific technique employed, whether it be immunoblotting, immunoprecipitation, or immunofluorescence microscopy.

Delineating the roles for overlapping genes is inherently difficult. As VP2 and VP3 possess the full region that corresponds to VP4, the membrane association and permeabilization properties associated with VP4 can also be expected to play a role in the functions of VP2 and VP3 (3, 8, 13). This VP4 region in the context of VP2 and VP3 likely contributes to viral entry and/or penetration, two steps that involve crossing membranes. The overlapping nature of the genes also makes it impossible to disrupt VP4 expression without affecting the sequence of VP2 and VP3, further complicating the separation of roles. We chose the most conserved substitution that involved a Met-to-Ile alteration, but the other 18 amino acids could also be tested for their effects. Interestingly, the identical alteration of the VP4 start Met was discovered using a temperature-sensitive screen to isolate mutants in SV40 (12). This independent study reports similar growth defects for the mutant, demonstrates that it was exacerbated in BS-C-1 cells, and states that bands were observed near the molecular weight of VP4 but VP4 could not be distinguished specifically. These authors also suggest that the phenotype may be due to an entry defect that is potentially caused by polar effects of the mutation on VP2 or VP3. It very well may be, but instead, we prefer to ask that scientists interested in this interpretation go and make their own assessment of these studies.
